# Synthesis of Multicolor Carbon Dots Catalyzed by Inorganic Salts with Tunable Nonlinear Optical Properties

**DOI:** 10.3390/ma17010042

**Published:** 2023-12-21

**Authors:** Xiaoqing Niu, Ruipeng Hou, Luo Zhang, Hongli Gao, Junzhou Hu

**Affiliations:** 1 Institute of Geography, Henan Academy of Sciences, Zhengzhou 450052, China; 2 Henan Provincial Key Laboratory of Nanocomposites and Applications, Institute of Nanostructured Functional Materials, Huanghe Science and Technology College, Zhengzhou 450006, China

**Keywords:** carbon dots, synthesis, nonlinear optical properties, salt catalyst

## Abstract

The nonlinear optical properties of carbon dots have been in the spotlight in recent years. In light of the complexity and diversity of factors affecting the nonlinear optical properties of carbon dots, how to reveal the origin and physical mechanism of the nonlinear optical properties of carbon dots accurately has become a problem. In this work, a template-free method was designed to prepare carbon dots via solid-phase reaction with phloroglucinol as a single carbon source and sodium bisulfate as the catalyst. This method is simple, green, safe, and easy to be prepared on a large scale. Three carbon dots with different luminous colors were obtained by simply adjusting the reaction temperature. The rise of reaction temperature affects the surface functional groups, and then hinders the luminescence of surface states, leading to the change of luminescence properties. The nonlinear optical properties of carbon dots were analyzed by the Z-scan technique. Surprisingly, all carbon dots have nonlinear optical responses, but there are differences in performance. Results prove the increase in sp^2^ domains may contribute to the significant improvement of the nonlinear optical properties of carbon dots, indicating a direction to improve the nonlinear optical properties of carbon dots.

## 1. Introduction

Carbon dots, as an emerging photoluminescent material, have been extensively applied in bioimaging, biosensors, light-emitting diodes, and catalysis [1,2,3,4,5]. Compared with semiconductor quantum dots, carbon dots have superior properties such as environmentally benign chemical composition, tunable photoluminescence properties, diverse surface groups, and unique photoelectric and physicochemical properties, which bring a bright prospect [6,7,8,9]. To date, there have been various methods to synthesize carbon dots, such as laser ablation [10,11], electrochemical oxidation [12,13], acid oxidation [14,15], hydrothermal/solvothermal method [16,17,18], pyrolysis [19], microwave [20,21], magnetic hyperthermia [22], plasma treatment [23], etc. Although carbon dots can be prepared from abundant carbon precursors by multiple inexpensive methods, it is still challenging to obtain carbon dots on a large scale. Firstly, in the industrial production of carbon dots, the traditional methods may produce a large amount of waste and thus increase the production costs. Moreover, the low yield is another major obstacle to the practical application of carbon dots. In addition, the complex and time-consuming post-process is also a crucial problem for industrialization production. Hence, it is pressing to develop simple and green methods to prepare carbon dots on a large scale.

The unique optical properties of carbon dots have been the concerns of researchers. The fluorescence of carbon dots is highly dependent on precursors, reaction conditions, and synthesis routes [24]. At present, the emission wavelength of carbon dots is available to be tuned from deep ultraviolet to near-infrared region by selecting different precursors [25,26,27,28]. Jiang et al. obtained blue, green, and red emissive carbon dots from three isomeric phenylenediamines via ethanol solvothermal treatment and concluded the size and nitrogen content of the carbon dots resulted in significant differences in luminescence properties [29]. Ding et al. prepared a series of carbon dots with different emission wavelengths ranging from 440 nm to 625 nm with p-phenylenediamine and urea as raw materials through hydrothermal synthesis and column chromatography separation. Subsequently, through detailed characterization, it was found the emission of carbon dots was not dependent on the particle size but controlled by the surface states. With the increase in the number of oxygen-containing groups on the surface of carbon dots, the emission wavelength was red-shifted [30]. Lu et al. synthesized near-infrared luminescent carbon dots with an emission wavelength of 710 nm through hydrothermal treatment of dopamine and o-phenylenediamine in acidic conditions and attributed the photoluminescence origin of carbon dots to the synergistic effect of carbon core and surface states [31]. However, the luminescence mechanism of carbon dots is still controversial due to the diversity of carbon dots and the complexity of their structures. What is more, most synthetic methods of carbon dots are liquid-phase synthesis. The involvement of different solvents makes the reaction environment more complex. Meanwhile, side reactions will occur, which will lead to the complexity of the structure and the heterogeneity of the surface states of carbon dots. It is a looming challenge to prepare carbon dots from a single carbon source via solid-phase methods to analyze the structures and properties of carbon dots and further reveal their luminescence mechanism more exactly.

Recently, besides photoluminescence and electrochemiluminescence properties, the nonlinear optical properties of carbon dots have also attracted much attention [32,33,34,35]. Bourlinos et al. first reported the nonlinear optical properties of carbon dots synthesized from lauryl gallate in 2013 [36]. By Z-scan analysis, it is found the organophilic carbon dots exhibited significant third-order nonlinear optical response comparable to that of fullerene. Aloukos et al. synthesized organophilic and hydrophilic carbon dots respectively, and discussed the difference in nonlinear optical response [37]. Tan et al. prepared carbon dots by femtosecond laser ablation of bagasse, and the resulting carbon dots had a strong optical limiting effect on the femtosecond laser pulse at 800 nm with a threshold of 74 mJ/cm^2^ [38]. Currently, the research on the nonlinear optical properties of carbon dots is just beginning, and considerable efforts are in demand to broaden the application fields of carbon dots.

Anhydrous sodium bisulfate, which has hygroscopic property, can be used as a reaction catalyst to promote the dehydration and condensation of the reaction precursor and the formation of carbon dots. Here, a template-free solid-phase synthesis method was designed using anhydrous sodium bisulfate as a catalyst and phloroglucinol as the single carbon source. By changing the reaction temperature, different luminescent carbon dots were obtained. The luminescence mechanism was discussed and the nonlinear optical properties of the carbon dots were measured in detail.

## 2. Materials and Methods

### 2.1. Preparation of Carbon Dots

A total of 0.2 g phloroglucinol (Aladdin, Shanghai, China) was dissolved in 4 mL of deionized water, heated, and stirred until it was completely dissolved, and then placed in an ice-water bath. After rapid stirring, a large amount of solid was precipitated, then filtered, and collected to obtain phloroglucinol nanocrystals. The phloroglucinol nanocrystal and sodium bisulfate (Aladdin, Shanghai, China) were ground evenly (the molar ratio of 1:1), and then transferred to a combustion boat for reaction at 200, 180, and 160 °C for 2 h. After the reaction, the solid was centrifuged by the mixed solution of deionized water and ethanol to remove salts and impurities in the reaction system [39]. The centrifuged sediment was dried in a vacuum oven (Jing Hong, Shanghai, China) at 60 °C. The reaction products at 200, 180, and 160 °C were named as sample A, sample B, and sample C, respectively.

### 2.2. Nonlinear Optical Measurement of Carbon Dots

**Figure 1:**
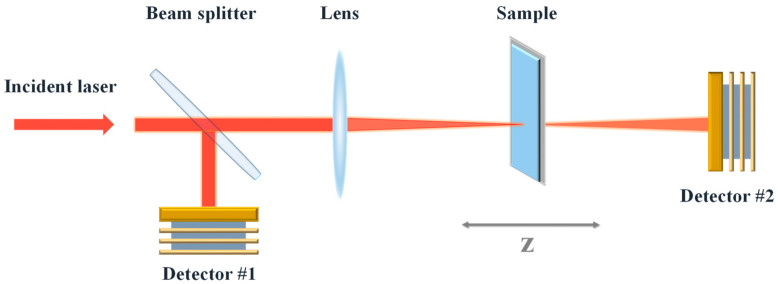
Illustration of an open-aperture Z-scan setup.

The nonlinear optical (NLO) properties of carbon dots were measured using a nano-second pulsed laser with a repetition rate of 10 Hz at a wavelength of 532 nm generated by the Q-switched YAG:Nd^3+^ laser (LOTIS TII, Minsk, Belarus) utilizing an open-aperture Z-scan technique. The ethanol solution of carbon dots (16 mg/mL) was added to a 1 mm-thick quartz cuvette placed on a moving platform and then moved along the *Z*-axis of the incident beam. Two energy detectors were used to measure the input energy and transmission energy. The two detectors were connected to the energy meter. The data were collected by a computer through the GPIB (Universal Interface Bus) interface, and the normalized transmittance was calculated. The open-aperture Z-scan setup is shown in the Figure 1.

## 3. Results and Discussion

**Scheme 1:**
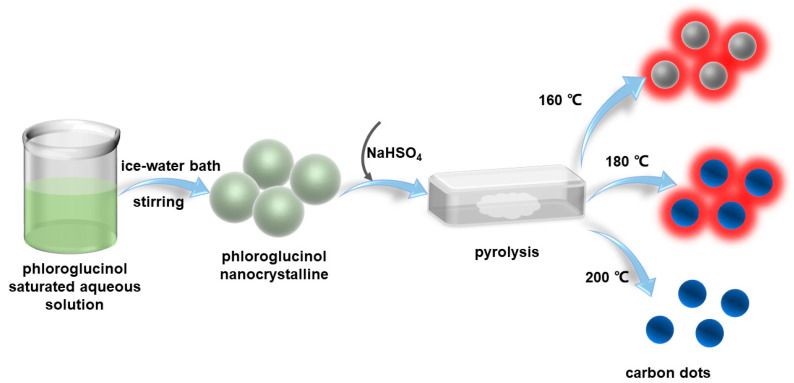
Schematic illustration of solid-phase synthesis of carbon dots by salt catalysis.

The solid-phase synthesis strategy of carbon dots was illustrated in Scheme 1. First, the saturated solution of phloroglucinol was transformed into uniform nanocrystals by stirring rapidly in an ice-water bath and extracting with filtration. Then the phloroglucinol nanocrystals were uniformly wrapped with sodium bisulfate, which could not only promote the formation of carbon dots, but also avoided the excessive aggregation of carbon dots. The sodium bisulfate was utilized as the pyrolysis-promoting agent [40], which not only played a role in dehydration in the process for carbon dots synthesis, but also accelerated the process of the reaction, contributing to forming a more stable lattice structure. Whereafter, two powders were blended well and heated under atmospheric pressure; the carbon dots were obtained following by deionized water and ethanol washing. We calculated the yields of the carbon dots from different temperatures respectively, and found the sample C had the highest yield, which could reach up to 80%. As shown in Figure S1, the carbon dots exhibit similar UV-Vis absorption properties. An obvious absorption peak at 560 nm was attributed to the surface states of the carbon dots [30]. With the rise of reaction temperature, the absorbance of the corresponding carbon dots at 560 nm was gradually weakened at the same concentration, which led to the final display of different fluorescence emission colors (Figure S2). Sample A emitted blue fluorescence with an emission peak of 460 nm while sample C showed red fluorescence, and the emission peak was at 600 nm. Meanwhile, sample B exhibited two emission peaks in blue and red regions. The fluorescence quantum yields of sample A, sample B, and sample C are 23.5%, 16.7%, and 12.6%, respectively. As indicated above, the reaction temperature has a significant effect on the fluorescence properties of the final product via this solid-phase synthesis method.

**Figure 2:**
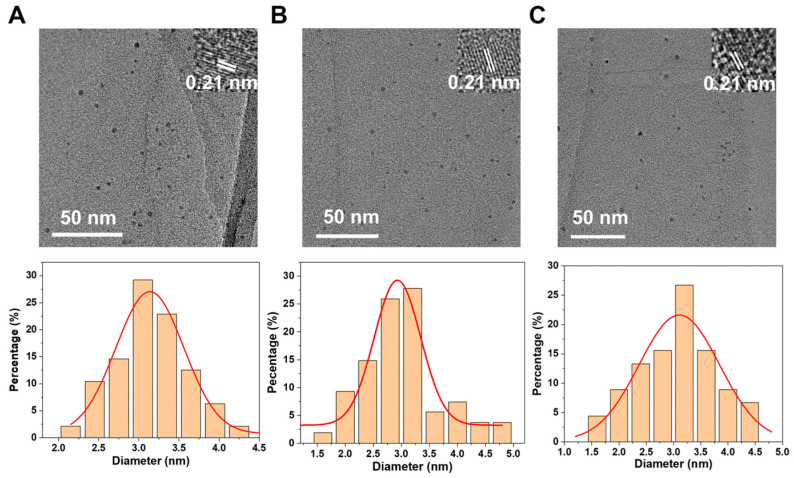
TEM images and corresponding particle size distribution of carbon dots prepared at (**A**) 200 °C (**B**) 180 °C (**C**) 160 °C, respectively.

To clarify the fluorescence origin of these carbon dots, the particle sizes and structures of the carbon dots were analyzed in detail. Figure 2 revealed these carbon dots were all monodisperse spherical nanoparticles with similar average diameters of 3 nm and had distinct lattice fringes with spacing of 0.21 nm corresponding to the (100) plane of the graphene [41], which indicated the particle size of carbon dots remained the same as the reaction temperature altered.

**Figure 3:**
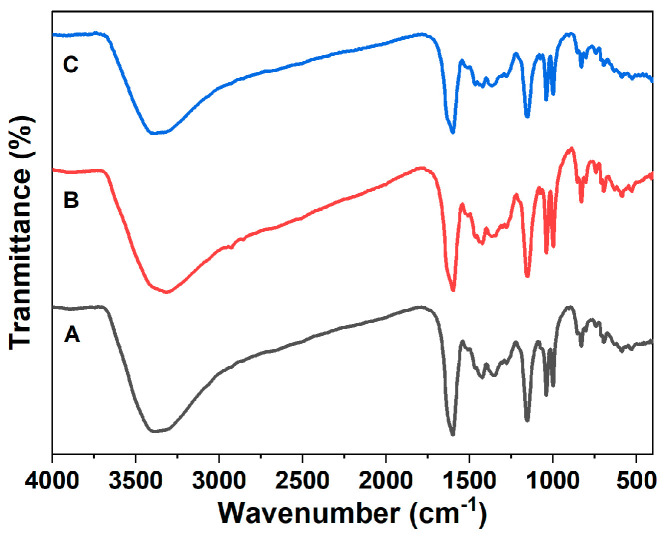
FTIR spectra of carbon dots prepared at (**A**) 200 °C (**B**) 180 °C (**C**) 160 °C, respectively.

**Figure 4:**
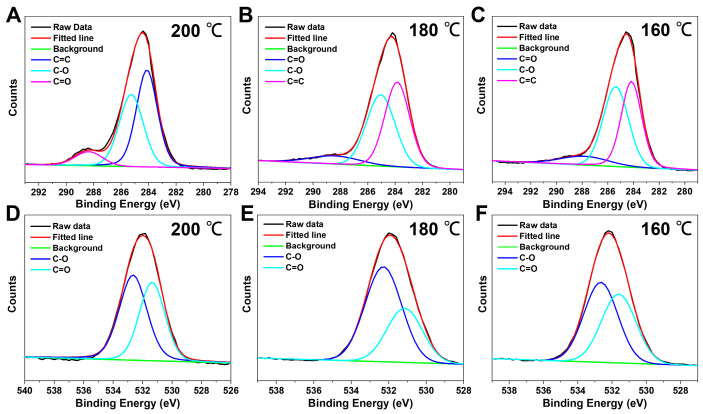
High resolution (**A**–**C**) C1s and (**D**–**F**) O1s spectra of carbon dots prepared at different temperatures.

The Fourier transform infrared (FTIR) spectra in Figure 3 presented the stretching vibration of O-H bond at 3400 cm^−1^, C=C bond at 1604 cm^−1^, and C-O bond at 1146 cm^−1^ in all the samples, showing the three samples had similar functional groups. X-ray photoelectron spectroscopy (XPS) measurements were performed to further unveil the surface structures of the carbon dots. As shown in Figure S3, two typical peaks of these carbon dots at 284 eV and 532 eV are observed, attributed to C1s and O1s, respectively, which indicate the carbon dots are all composed of carbon and oxygen elements without heteroatom doping caused by the presence of sodium bisulfate. C1s high-resolution spectra (Figure 4) of the carbon dots were fitted into three peaks, which were assigned to C=C bond, C-O bond, and C=O bond, respectively. O1s high-resolution spectra (Figure 4) were deconvoluted with two peaks corresponding to the C-O bond and the C=O bond, respectively.

When the reaction temperature decreased from 200 ℃ to 160 ℃, the content of oxygen element in the carbon dots increased slightly, from 26.90% to 28.10%, as estimated by XPS elemental analysis in Table 1, which indicated high temperature was unfavorable to oxygen-containing functional groups. Most importantly, the content of the C=C bond in the carbon dots changed significantly. From sample A to sample C, the content of the C=C bond decreased from 50.38% to 43.35%, while the content of the C-O bond and C=O bond both showed an increasing trend, which was consistent with the FTIR results.

**Scheme 2:**
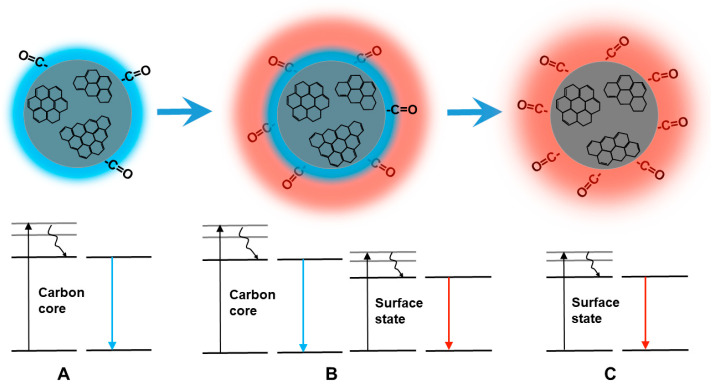
Schematic illustration of the luminescence mechanism of (**A**) sample A (**B**) sample B and (**C**) sample C. (Color arrows represent the corresponding emission light generated by the sample).

We also collected the time-resolved photoluminescent spectra of these carbon dots. The fluorescence decay curves of the carbon dots (Figure S4) all exhibited double exponential decay. The shorter fluorescence lifetime comes from the surface state, and the longer fluorescence lifetime is derived from the sp^2^ conjugated domain of the carbon core [42]. According to the fitting results (Table 2), from sample A to sample C, the average fluorescence lifetime of the carbon dots was 4.56 ns, 4.32 ns, and 4.29 ns, respectively. Moreover, the proportion of short lifetime component was gradually increased, while the proportion of long lifetime component was cut down, which was caused by the increasing content in C-O bond and C=O bond and the decreasing content in C=C bond from sample A to sample C as shown in FTIR and XPS results. Hence, it is supposed the shortening of the fluorescence lifetime of the carbon dots was significantly related to the surface states. Although there is no definite understanding of the fluorescence mechanism of carbon dots, a large amount of evidence indicates the fluorescence behavior of carbon dots is mainly related to the conjugated domains and surface states [43,44,45,46,47,48]. Combined with the above structural analysis and spectral analysis, it was concluded the optical properties of the carbon dots were affected by both the carbon core and the surface states. These carbon dots prepared by this method contain two emission centers, as shown in Scheme 2, which originate from the sp^2^ conjugated domain of carbon core (C=C bond) and surface states (oxygen-containing functional groups), respectively. Since the surface oxidation of carbon dots could lead to the reduction of band gap and the red-shift of emission wavelength, short wavelength emission (blue fluorescence) was ascribed to the sp^2^ conjugated domain of the carbon core, while long wavelength emission (red fluorescence) was attributed to the surface states. As the reaction temperature altered, the dominant emission centers changed accordingly. As a result, the carbon dots showed different fluorescence properties. At low temperature (160 °C), the short lifetime component predominated, so the sample C showed red fluorescence from the surface states. When the reaction temperature rose to 200 °C, high temperature destroyed oxygen-containing functional groups and thus hindered the luminescence from surface states. As can be seen from the fluorescence decay curves, the emission proportion of short-life carbon dots obtained at 200 ℃ is small. Simultaneously the structure of carbon core becomes more stable due to the high-temperature treatment, and the content of the C=C bond increases. Therefore, the emission of sample A is mainly from the blue fluorescence in the sp^2^ conjugated domain of carbon core.

**Figure 5:**
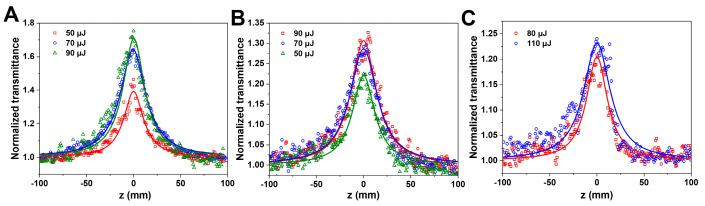
Open-aperture Z-scan results of (**A**) sample A (**B**) sample B and (**C**) sample C under 532 nm laser excitation.

As is well-known, the amount of sp^2^ carbon (C=C) versus sp^3^ hybrid carbon inside the core of the carbon dots, which determines whether the carbon core is crystalline or amorphous, and the functional groups on the surface of the carbon dots, can vary the energy gap and have a significant influence on the optical properties of the carbon dots accordingly [49]. This makes it a very difficult task to explore the nonlinear optical properties of different kinds of carbon dots in depth. Herein, we used the same method to synthesize three carbon dots with the same size and similar structure but different luminescence properties by just changing the reaction temperature, and discussed the differences of the nonlinear optical properties of these carbon dots and the influencing factors. The Z-scan technique is one of the most common techniques for nonlinear optical measurement because of its simple operation and high sensitivity. A nanosecond laser light source (excitation source: 532 nm, 12 ns, 10 Hz) was utilized to examine the nonlinear optical properties of the ethanol solution of these carbon dots by the open-aperture Z-scan technique. Figure 5 showed the results of the normalized transmittance of the open-aperture Z-scan curves of three carbon dots excited by 532 nm lasers of different energies. Under the excitation of different energy pulses, each curve presented an upward sharp narrow peak located at the beam focus. Since the solvent (anhydrous ethanol) had no nonlinear optical properties, it could be concluded that the saturated absorption behavior derived from the carbon dots.

The following equation was used to fit the Z-scan data, and the nonlinear absorption coefficient *β*_eff_ of the samples under different energies was obtained from the fitting results.

(1) $$\frac{dI\left(z^{\prime }\right)}{dz^{\prime }}=-\alpha \left(I\right)$$

(2) $$\alpha \left(I\right)=\alpha_{0}+\beta_{\mathrm{eff}}I$$

(3) $$T_{\mathrm{NL}}(z)=\sum_{m=0}^{\infty }\frac{{\left[-q_{0}\left(z\right)\right]}^{m}}{{\left(m+1\right)}^{3/2}}$$

(4) $$q_{0}\left(z\right)=\frac{\beta_{\mathrm{eff}}I_{0}L_{\mathrm{eff}}}{1+{\left(\frac{z}{z_{0}}\right)}^{2}}$$

(5) $$L_{eff}=\frac{1-e^{-\alpha_{0}L}}{\alpha_{0}}$$

where I is the intensity of the incident laser, $I_{0}$ is the intensity of the light at the focus of the incident axis, $z^{\prime }$ is the propagation distance in the sample, z is the position of the sample, $\alpha_{0}$ is the linear absorption coefficient of the sample, $z_{0}=\pi \omega_{0}^{2}/\lambda$ is the diffraction length, $L_{\mathrm{eff}}$ is the effective length of the sample, L is the path length of the sample. According to the equations, the nonlinear optical parameters of these carbon dots were calculated, and the results were shown in Table 3. With the increase in laser energy, the nonlinear absorption performance of carbon dots was gradually enhanced. Under the same energy of laser irradiation (27.09 MW cm^−2^), the nonlinear absorption coefficients of sample A and sample B were −183.98 cm GW^−1^ and −82.02 cm GW^−1^, respectively, while sample C had no obvious nonlinear optical response. Sample C exhibited the nonlinear optical response only under the excitation of higher laser energy, and the nonlinear absorption coefficient was −46.26 cm GW^−1^ (power density was 43.34 MW cm^−2^). As the laser energy increased, the nonlinear absorption coefficient of sample A changed significantly from −183.98 cm GW^−1^ to −246.55 cm GW^−1^, while the nonlinear optical responses of sample B and sample C were not improved evidently. In addition, the third-order nonlinear optical quality factor (FOM) was defined to evaluate the saturated absorption performance analysis of carbon dots, in which FOM = |Imχ^(3)^/α_0_|, where Imχ^(3)^ is the imaginary part of third-order nonlinear polarizability and directly related to the nonlinear absorption coefficient *β*_eff_. According to the data in Table 3, the corresponding FOM values of sample A and sample B under laser excitation of the same energy (27.09 MW cm^−2^) were 2.84 × 10^−12^ and 2.57 × 10^−12^ esu cm, respectively. The FOM value of sample C excited by a higher energy laser was also much smaller than that of sample A. These results demonstrated the nonlinear optical property of the carbon dots was enhanced with rising reaction temperature. Combined with XPS results of the carbon dots, it can be deduced as the reaction temperature rises, the content of sp^2^ carbon atoms in the carbon dots increases, and the Pauli-blocking of electrons in the sp^2^ conjugated domain also gradually increases [50,51]. Therefore, the saturation absorption effect grows obviously. In conclusion, the sp^2^ conjugated domain of carbon dots plays an important role in their nonlinear optical response, and the carbon dots with more sp^2^ domains are expected to have greater nonlinear optical properties.

## 4. Conclusions

In summary, a simple template-free solid-phase synthesis method for carbon dots was developed with the advantages of low cost, convenient operation, safety and environmental friendliness, and the highest yield of carbon dots reached 80% via this method. These carbon dots have similar particle size and lattice structure, but different luminescence characteristics, which indicates the size of carbon dots is not the main factor affecting the luminescence properties. It is proposed these carbon dots have two emission centers, namely carbon core and surface states. With the change of temperature, the leading emission center changes, and accordingly, the emission color of carbon dots also changes. The nonlinear optical results demonstrated these carbon dots all had nonlinear optical responses, but the nonlinear absorption coefficient was different, which may be caused by the different number of sp^2^ carbon atoms. This provides an idea for the nonlinear optical performance improvement of carbon dots, and broadens the potential applications of carbon dots, which is expected to be used as a saturated absorber in mode-locked and Q-switched lasers.

## Figures and Tables

**Table 1:** XPS data analysis of carbon dots prepared at different temperatures.

Sample	C	O	C=C	C-O	C=O
A	73.10%	26.90%	50.38%	40.86%	8.73%
B	71.64%	28.36%	47.09%	44.17%	8.74%
C	71.90%	28.10%	43.35%	46.87%	9.78%

**Table 2:** Fluorescence lifetimes of carbon dots prepared at different temperatures.

Sample	τ_1_ (ns)	A_1_ (%)	τ_2_ (ns)	A_2_ (%)	τ_ave_ (ns)	R^2^
A	1.03	40.71	5.05	59.29	4.56	0.999
B	2.65	65.88	5.80	34.12	4.32	0.999
C	3.02	72.93	6.01	27.07	4.29	0.999

**Table 3:** Nonlinear optical parameters of the carbon dots.

Sample	I (MW cm^−2^)	*β*_eff_ (cm GW^−1^)	Imχ^(3)^ (esu)	FOM (esu cm)
A	27.09	−183.98	−6.97 × 10^−11^	2.84 × 10^−12^
	37.88	−246.55	−9.34 × 10^−11^	3.80 × 10^−12^
B	27.09	−82.02	−3.11 × 10^−11^	2.57 × 10^−12^
	37.88	−90.96	−3.45 × 10^−11^	2.85 × 10^−12^
C	43.34	−46.26	−1.75 × 10^−11^	1.36 × 10^−12^
	59.58	−47.90	−1.81 × 10^−11^	1.41 × 10^−12^

## Data Availability

Data are contained within the article.

## Supplementary Materials

The following supporting information can be downloaded at: https://www.mdpi.com/article/10.3390/ma17010042/s1, Figure S1: UV-Vis absorption spectra of carbon dots prepared at (A) 200 °C (B) 180 °C (C) 160 °C, respectively; Figure S2: Fluorescence emission spectra of carbon dots prepared at (A) 200 °C (B) 180 °C (C) 160 °C, respectively; Figure S3: XPS spectra of carbon dots prepared at (A) 200 °C (B) 180 °C (C) 160 °C, respectively; Figure S4: Time-resolved PL spectra of carbon dots prepared at (A) 200 °C (B) 180 °C (C) 160 °C, respectively.
