# Supplementary material for: Synthesis of Multicolor Carbon Dots Catalyzed by Inorganic Salts with Tunable Nonlinear Optical Properties
Source: Materials (Basel). 2023 Dec 21;17(1):42. doi: 10.3390/ma17010042 (PMC10779595; doi:10.3390/ma17010042)
Supplement: Supplementary file 1 [file materials-17-00042-s001.zip › materials-2747616-supplementary.pdf]

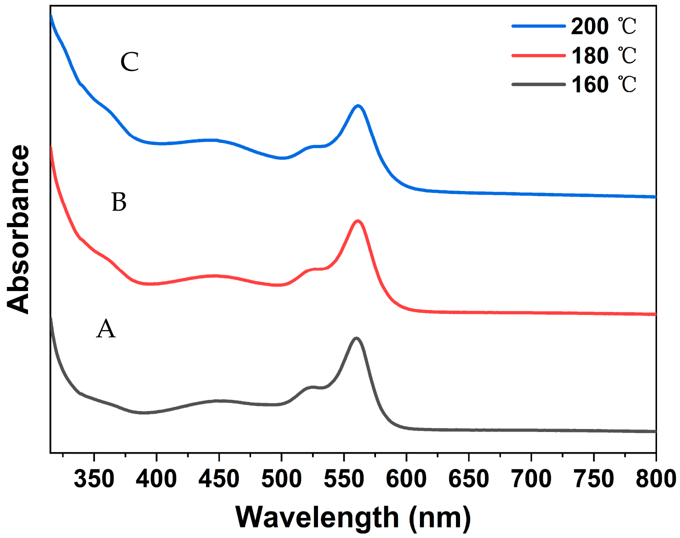

Figure S1: UV-Vis absorption spectra of carbon dots prepared at (A) 200 ° C (B) 180 ° C (C) 160 ° C, respectively.

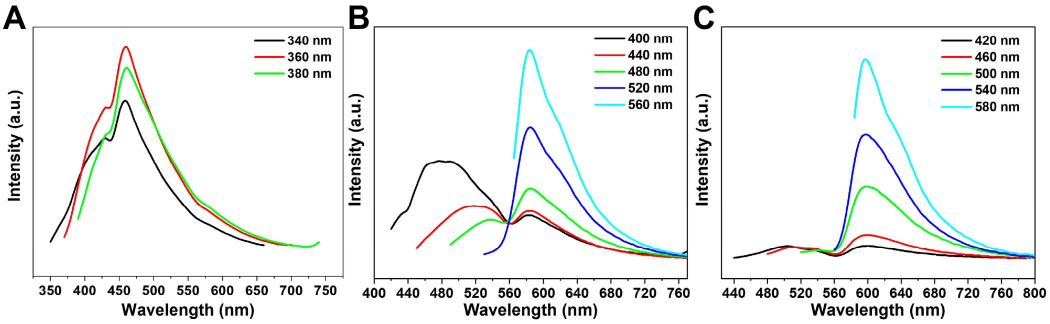

Figure S2: Fluorescence emission spectra of carbon dots prepared at (A) 200 ° C (B) 180 ° C (C) 160 ° C, respectively.

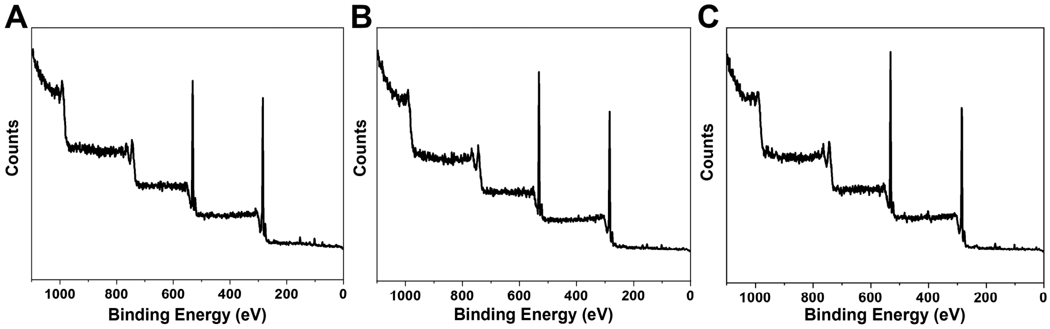

Figure S3: XPS spectra of carbon dots prepared at (A) 200 ° C (B) 180 ° C (C) 160 ° C, respectively

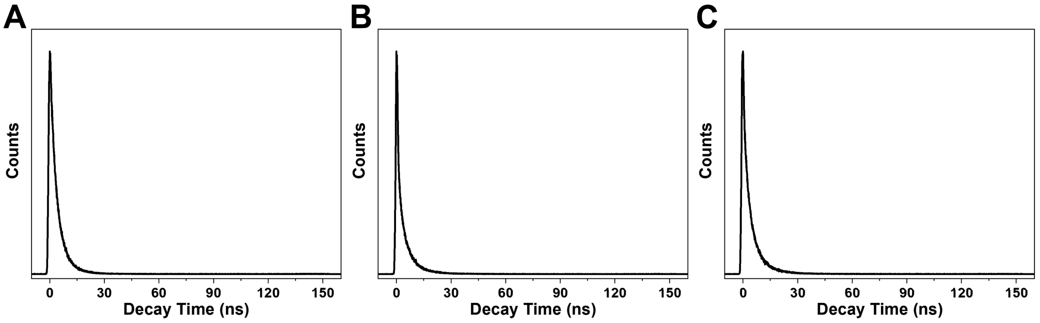

Figure S4: Time-resolved PL spectra of carbon dots prepared at (A) 200 ° C (B) 180 ° C (C) 160 ° C, respectively.
